# Plasmacytoid dendritic cell interferon-α production to R-848 stimulation is decreased in male infants

**DOI:** 10.1186/1471-2172-13-35

**Published:** 2012-07-06

**Authors:** Jennifer P Wang, Lei Zhang, Rachel F Madera, Marcia Woda, Daniel H Libraty

**Affiliations:** 1 Division of Infectious Disease and Immunology, Department of Medicine, University of Massachusetts Medical School, 364 Plantation, Worcester, MA, 01605, USA

**Keywords:** pDC, IFN-α, TNF-α, Infant, TLR7

## Abstract

**Background:**

Sex differences in response to microbial infections, especially viral ones, may be associated with Toll-like receptor (TLR)-mediated responses by plasmacytoid dendritic cells (pDCs).

**Results:**

In this study, we identified sex differences in human infant pDC interferon-α production following challenge with the TLR7/8 agonist R-848. Male pDC responses were significantly lower than those of females during early infancy. This difference may be attributed to the androgen surge experienced by males during the early infancy period. Pretreatment of human pDCs with dihydrotestosterone produced a significant reduction in interferon-α production following R-848 challenge.

**Conclusions:**

Androgen-mediated regulation of pDC TLR7-driven innate immune responses may contribute to the observed sex differences in response to infections during early infancy.

## Background

Plasmacytoid DCs (pDCs) are highly specialized cells that produce large amounts of interferon-α (IFN-α) in response to a wide range of viruses and other microbial stimuli [1,2]. Infant and adult human pDCs express high levels of Toll-like receptors (TLRs) 7 and 9, but not TLR8 [3,4]. Human TLRs 7 and 8 are members of the endosomal TLR9 subfamily that sense RNA moieties intracellularly and signal through the adaptor MyD88 [5]. The imidazoquinolone and guanosine-like compound, resiquimod (R-848), stimulates human TLRs 7/8 [6,7].

Sex differences in adult pDC IFN-α production have been previously described, with production being higher in females than males [8,9]. However, the reported effects of female sex hormones on TLR7-mediated pDC IFN-α production have been varied [8-10]. The effects of male sex hormones have not been previously determined. Within the first six months of life, infants experience a “mini-puberty”. During this time period, circulating levels of sex hormones approach the levels seen during puberty [11]. We found that during the “mini-puberty” period female infant pDCs also have higher pDC IFN-α production than male infants in response to 1 μM R-848 stimulation. Androgen signaling downregulated R-848 stimulated pDC IFN-α production. This sex effect on pDC IFN-α production may play a role in the female survival benefit seen during early infancy. However, the effect of sex on pDCs may have a cost of higher rates of systemic lupus erythematosus (SLE) and autoimmune diseases later in adult females compared to adult males [12,13]. Here we describe a novel effect of male sex hormones on TLR7-mediated pDC IFN-α production.

## Results and discussion

**Figure 1:**
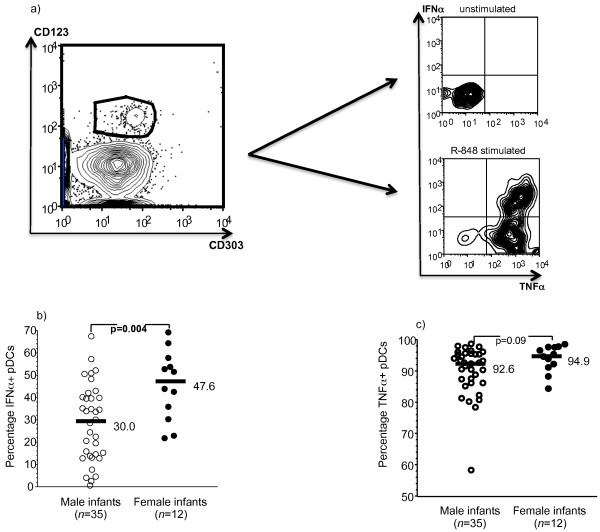
**Intracellular cytokine staining for IFN-α and TNF-α in pDCs upon 1 μM R-848 stimulation. a** pDCs were gated as CD123^+^CD303^+^ cells in infant PBMC (left column) after selection for live (Live/Dead Aqua -), single, and large mononuclear cells. All IFN-α producing pDCs upon 1 μM R-848 stimulation also produced TNF-α (right column). One representative example of the gating strategy in infant PBMC is shown. **b** The percentage of IFN-α + pDCs, **c** but not TNF-α + pDCs, is higher in female infants compared to male infants upon 1 μM R-848 stimulation. Infant age = 2.1 [1.6-3.8] months (median [95 % CI]). Unstimulated background is subtracted from the values. Bars = median values. Comparisons were performed using the non-parametric Mann Whitney U test. P-values < 0.05 were considered significant.

As part of a prospective study of dengue virus infections during infancy in San Pablo, Philippines, we collected peripheral blood mononuclear cells (PBMC) from healthy infants at approximately two months of age. We measured pDC IFN-α and tumor necrosis factor-α (TNF-α) production to 1 μM R-848 stimulation by intracellular cytokine staining (ICS) in healthy infant PBMC from 47 randomly selected donors (ages = 2.1 [1.6-3.8] months). Figure 1a shows the gating strategy for the flow cytometry. pDCs were 2.2 [0.8-6.2]% of live PBMC in our early infancy samples. We found that the percentage of IFN-α + pDCs (Figure 1b), but not TNF-α + pDCs (Figure 1c), was significantly higher in female infants compared to male infants following 1 μM R-848 stimulation. There were no significant differences in age or World Health Organization (WHO) anthropometric measures between the sexes (weight-for-age, length-for-age, weight-for-length, or body mass index-for-age z scores). PBMC viability was measured by trypan blue exclusion in the cell cultures and was similar between the two sexes (data not shown). Higher R-848 and TLR7-mediated pDC IFN-α production has been previously reported in adult females compared to adult males [8,9]. To the best of our knowledge, this is the first time a similar phenomenon has been reported during early infancy. TLR7 expression has not been found to be different between the sexes [8]. In one report, 17-β-estradiol was reported to augment TLR9-mediated pDC IFN-α production [14]; but in another report, 17-β-estradiol did not affect TLR7-mediated pDC IFN-α production [8]. The reported effects of progesterone on TLR-mediated pDC IFN-α production have also been conflicting. Meier et al. demonstrated that plasma progesterone levels significantly correlated with pDC IFN-α production in response to stimulation with an HIV-1–derived TLR7/8 ligand [9]. By contrast, Hughes et al. found that progesterone inhibited CpG-mediated and vesicular stomatitis virus-induced IFN-α production by pDCs [10].

**Figure 2:**
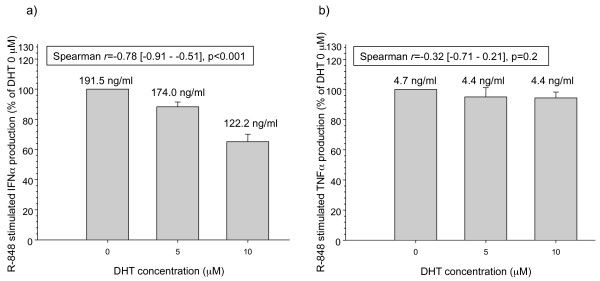
**The androgen dihydrotestosterone (DHT) decreases pDC R-848-stimulated IFN-α production, but not TNF-α, in a dose-dependent manner. IFN-α and TNF-α production were measured by ELISA in cell-free culture supernatants from 5 × 10**^**4 **^**purified healthy adult female pDCs/well.** pDC viability was measured by trypan blue exclusion and was similar in all groups (data not shown). **a** pDC IFN-α production due to R-848 1 μM stimulation; *n* = 5 independent experiments (% of pDC IFN-α production at DHT 0 μM). **b** pDC TNF-α production due to R-848 1 μM stimulation; *n* = 4 independent experiments (% of pDC TNF-α production at DHT 0 μM). Bar values and error bars are mean ± S.E.; Spearman *r* values and [95 % confidence intervals] are shown. Numbers above bars are mean cytokine supernatant levels (ng/ml). P-values < 0.05 were considered significant.

During the “mini-puberty” stage [11], male infants have markedly higher circulating testosterone and dihydrotestosterone (DHT) levels compared to female infants [15]. We therefore hypothesized that androgen signaling would decrease pDC IFN-α production in response to R-848 stimulation. DHT is a more potent androgen than testosterone [16]. Pretreatment of female adult pDCs with DHT decreased R-848-stimulated IFN-α production, but not TNF-α production, in a dose dependent manner (Figure 2). A similar finding was seen for CpG-mediated TLR9 stimulation in the pDCs (data not shown). The downregulation of TLR7 and TLR9 stimulated pDC IFN-α production contrasts with the reported positive associations between androgens and serum inflammatory markers [17]. pDC IFN-α production is dependent on interferon regulatory factor 7 (IRF7) [18]. The IRF7 promoter contains androgen responsive elements and higher levels of IRF7 have been seen in the lungs of female Norway rats compared to males [19]. We postulate that the lower R-848 stimulated pDC IFN-α production in male infants is at least partly due to androgen effects on IRF7 levels within the first six months of life. Future studies are planned to examine the effect of androgens on pDC IRF7 levels.

Female infants enjoy a survival benefit compared to male infants in the first six months of life. In particular, *Staphylococcus aureus* infections [20] and respiratory viral infections such as respiratory syncytial virus (RSV) [21] predominate in males during early infancy. *Staphylococcus aureus* and several airborne and respiratory RNA viruses (including RSV) can modulate TLR7-mediated pDC IFN-α production during their pathogenesis [22-24]. Higher pDC IFN-α production in females during early infancy may contribute to their survival benefit. Later in life, this sex effect may contribute to a higher incidence of SLE and autoimmune diseases in females [12,13].

## Conclusions

We found that R-848-stimulated pDC IFN-α production, but not pDC TNF-α production, was higher in girls compared to boys during early infancy. Androgen signaling downregulated R-848-stimulated pDC IFN-α production, but not pDC TNF-α production, in a dose dependent fashion. We postulate that androgen effects on pDCs play a role during the “mini-puberty” of early infancy in the sex differences of R-848-stimulated pDC IFN-α production.

## Methods

### Infant clinical study

The infant clinical study was approved by the institutional review boards of the Research Institute for Tropical Medicine, Philippines, and the University of Massachusetts Medical School (UMMS). Mothers and their healthy infants were recruited and enrolled after providing written informed consent. Study enrollment began in October 2006 in San Pablo, Philippines. Blood samples were collected from healthy infants and their mothers when the infant was between 6–18 weeks old. Normalized child growth indicators were determined using WHO child growth standards [25]. PBMC were isolated by Ficoll-Hypaque density centrifugation from infant blood samples and cryopreserved.

### Antibodies and fluorophores

The following monoclonal antibodies (mAbs) and fluorophores were used: mouse mAb anti-human CD123 eFluor NC650 (eBioscience, San Diego, CA), anti-human CD303 APC (Miltenyi Biotec, Auburn, CA), anti-human tumor necrosis factor-α (TNF-α) PerCPCy5.5 (Biolegend, San Diego, CA), and anti-human IFN-α FITC (PBL Interferon Source, Piscataway, NJ).

### Intracellular cytokine staining (ICS)

PBMC were resuspended in RPMI 1640 (Invitrogen Life Technologies, Grand Island, NY), 10 % FCS (HyClone, Logan, UT), 10 ng/ml rIL-3 (R&D Systems, Minneapolis, MN), and 20 μg/ml DNase (Sigma-Aldrich, St. Louis, MO). 1.5 × 10^6^ PBMC were placed in polypropylene tubes with 1 μM R-848 (Invivogen, San Diego, CA 0.5 μg/ml brefeldin A (BD Pharmingen, San Diego, CA), and incubated for 16 h in a 37 °C/5 % CO_2_ incubator. The PBMC were then stained with Live/Dead Aqua (Invitrogen Life Technologies, Grand Island, NY), fixed and permeabilized (Invitrogen Life Technologies), and stained for pDC markers (CD123 and CD303) and cytokine production (IFN-α and TNF-α). Flow cytometry data was acquired on a FACSAria (BD Biosciences, San Diego, CA).

### Cell culture and ELISAs

Human pDCs were isolated from the blood of healthy adult donors under a protocol approved by the UMMS Institutional Review Board. PBMC were isolated using Ficoll-Hypaque density centrifugation, and pDCs were positively selected from the PBMC using magnetic beads (Miltenyi Biotec, Auburn, CA). 5 × 10^4^ pDCs were cultured in 96-well plates in 200 μl RPMI 1640, 10 % FCS, and 10 ng/ml rIL-3. 1 μM R-848 or 7.5 μg/ml CpG 2336 (Coley Pharmaceuticals, Wellesley, MA) was added to pDC cultures overnight in a 37 °C/5 % CO_2_ incubator; cell-free culture supernatants were collected for ELISAs at 18–24 h. IFN-α and TNF-α ELISAs (R&D Systems) were performed per the manufacturer’s instructions. All samples were assayed in duplicate. In some experiments, pDCs were pretreated for 1 h with dihydrotestosterone (DHT, Sigma-Aldrich) at the indicated concentrations.

### Statistical analysis

The SPSS software package (version 19.0) was used for statistical analyses. Comparisons between two groups were performed using a two-tailed Mann–Whitney U test. Spearman’s correlation was also determined. P < 0.05 was considered significant. Values are presented as the median [95 % confidence interval (CI)].

## Abbreviations

CI, Confidence interval; DHT, Dihydrotestosterone; ICS, Intracellular cytokine staining; IFN-α, Interferon-α; IRF7, Interferon regulatory factor 7; mAbs, Monoclonal antibodies; PBMC, Peripheral blood mononuclear cells; pDC, Plasmacytoid dendritic cell; RSV, Respiratory syncytial virus; SLE, Systemic lupus erythematosus; TLR, Toll-like receptor; TNF-α, Tumor necrosis factor-α; UMMS, University of Massachusetts Medical School; WHO, World Health Organization.

## Competing interests

There are no competing interests.

## Authors’ contributions

JPW- analyzed and interpreted data, wrote the manuscript. LZ- performed research, collected data. RFM- analyzed and interpreted data, wrote the manuscript. MW- performed research, collected data. DHL- designed research, performed statistical analysis, analyzed and interpreted data, wrote the manuscript. All authors read and approved the final manuscript.
